# miRNA-1175 downregulates a long non-coding natural antisense RNA and promotes long term memory

**DOI:** 10.1038/s41598-025-22550-w

**Published:** 2025-11-05

**Authors:** Sergei A Korneev, Gabriella Taylor, Owen S. Wells, Souvik Naskar, Elena I. Korneeva, Ildikó Kemenes, György Kemenes

**Affiliations:** 1 School of Life Sciences, University of Sussex, Brighton, BN1 9QG UK; 2 Department of Infectious Disease, Imperial College, London, SW7 2AZ UK

**Keywords:** Long non-coding RNAs, Natural antisense transcripts, Long-term memory, MiRNA, Nitric oxide synthase, Brain, Molecular biology, Neuroscience

## Abstract

Single-trial induced long-term memories (LTMs) require fast removal of inhibitory memory constraints following learning. We propose that the interplay between short non-coding RNAs (sncRNAs) and long non-coding RNAs (lncRNAs) is engaged in this process, suggesting the existence of a new and unexpected pathway for LTM formation. In the mollusc *Lymnaea*, this pathway involves the functional interaction between a typical sncRNA, Lym-miR-1175, and a lncRNA, *Lym-NOS1AS*. In the current study, we validate this hypothesis and provide evidence that: (1) LTM formation in *Lymnaea* is associated with the timed and targeted changes in the expression of Lym-miR-1175; (2) Lym-miR-1175 is a negative regulator of the *Lym-NOS1AS*; (3) Lym-miR-1175 is required for single-trial induced LTM; (4) Lym-miR-1175 and *Lym-NOS1AS* are co-expressed in a key neuron of the *Lymnaea* memory network. Thus, our data indicate an important role of the interaction between a miRNA and a lncRNA in one-trial learning.

**Supplementary Information:**

The online version contains supplementary material available at 10.1038/s41598-025-22550-w.

## Introduction

Both our everyday experience and the numerous behavioural studies confirm the general importance of repetition for the formation of enduring memories^1^. But sometimes a single but highly salient event also triggers long-term memory (LTM). Examples of this type of memory include “flash-bulb” memories in humans and associative memories for strongly aversive or highly rewarding stimuli in animals^2,3^. For everyday human experience even ordinary (i.e., non-flash-bulb type) single trial memories are much more relevant than those trained by repetition. For example, episodic memories forming after just one trial, or single-trial visual discrimination or social learning are probably more relevant to human behavior than most models based on conditioning and repetition^4^. However, it is not well understood, either in humans or animals, how a single episode of learning can gain immediate access to the molecular machinery responsible for LTM formation. New insights that can help to answer this question have emerged from the recent discovery that small non-coding RNAs (sncRNAs), including microRNAs (miRNAs), participate in the regulation of some important processes in the brain^5,6^. For example, it was reported that miRNAs are involved in the regulation of long-term synaptic plasticity^7–10^. Recently, we have extended these studies by addressing the question of whether miRNAs are engaged in the consolidation of LTM after single-trial conditioning. Using a well-established molluscan model organism, *Lymnaea stagnalis*^11–14^, we identified several miRNAs exhibiting training-induced alterations in their expression (hereafter LTM-related miRNAs). Of these LTM-related miRNAs, we have already characterised Lym-miR-137. We demonstrated that this miRNA down-regulates *Lym-CREB2* mRNA and thus removes an important molecular constraint of memory consolidation^15^. Interestingly, however, blockage of the Lym-miR-137 did not completely suppress memory formation indicating that there are other, as-yet-unknown memory constraints. Hence, we focused on Lym-miR-1175, which is also an example of the LTM-related miRNAs. Intriguingly, this miRNA has a putative target sequence located within a long non-coding RNA (lncRNA) called *Lym-NOS1AS*^16^. This lncRNA is transcribed from the non-template strand of the *NOS1* (*Nitric Oxide Synthase 1*) locus and belongs to a subfamily of lncRNAs known as long Natural Antisense Transcripts (long NATs). Notably, it was reported that the NOS-related long NATs act as negative regulators of *NOS* gene expression^17–22^. These observations are important because NOS protein catalyses the production of nitric oxide (NO), which is required for memory formation^23^. Taken together, these findings predict the existence of a new pathway for single-trial induced LTM formation, which involves the functional interaction between the two different classes of non-coding RNAs (ncRNAs), namely a typical sncRNA, Lym-miR-1175, and a long NAT, *Lym-NOS1AS*.

In this study, we provide support to this hypothesis by presenting the following findings: (i) our quantitative experiments reveal that single-trial reward conditioning leading to LTM formation is associated with the timed and targeted changes in the expression of Lym-miR-1175, (ii) using gain-of-function and site-directed mutagenesis experiments we demonstrate that Lym-miR-1175 is a negative regulator of the *Lym-NOS1AS* NAT, (iii) by employing an in vivo loss-of-function approach we provide evidence that Lym-miR-1175 is required for single-trial induced LTM, (iv) using in-situ hybridization and RT-PCR, we show that Lym-miR-1175, *Lym-NOS1AS* NAT and *Lym-NOS1* mRNA are co-expressed in the identified cerebral giant cells (CGCs), which are key neurons of the *Lymnaea* memory network^24,25^.

## Results

### Single-trial reward conditioning induces the timed and targeted differential regulation of Lym-miRNA-1175 in the learning ganglia

**Fig. 1:**
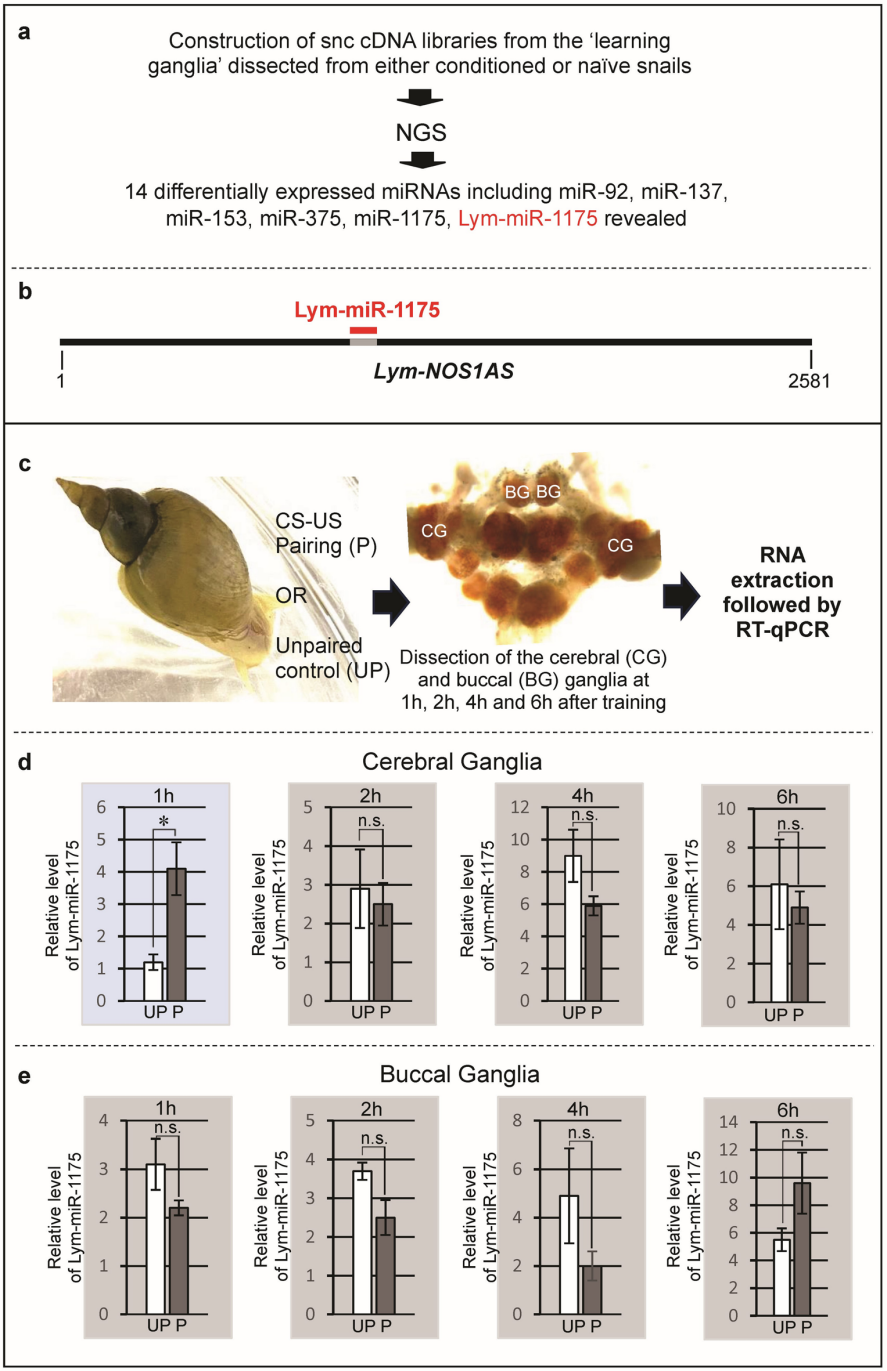
A LTM-related Lym-miRNA-1175 exhibits specific training-induced changes in its expression. (**a**) Schematic representation of the experiment to identify miRNAs involved in single-trial reward conditioning. (**b**) A schematic diagram showing that Lym-miR-1175 (red) has a putative target site (grey) located within the *Lym-NOS1AS* (diagram not drawn to scale). (**c**) Schematic representation of the experiment conducted to investigate whether single-trial reward conditioning is associated with specific changes in Lym-miR-1175 expression. A single pairing of amyl acetate, a conditioned stimulus (CS) and sucrose, an unconditioned stimulus (US) was employed to produce LTM. (**d**, **e**) Results of quantitative RT-PCR performed on the cerebral and buccal ganglia, respectively. The relative levels of Lym-miRNA-1175 expression in ganglia from unpaired control (UP, white bars, the CS and the US were separated by an interval of 1 h) and conditioned (paired, P, dark grey bars) animals dissected at 1 h, 2 h, 4 h and 6 h after training were calculated as 2^−ΔΔCt^. All data in this figure are shown as means ± SEM (*n* = 20 in each group, collected in 5 tubes each containing 4 either BG or CG). Asterisk indicates statistically significant difference (*p* = 0.036, t = 2.85, df = 4.87, unpaired two-tailed t-test with Welch’s correction) between the ‘conditioned’ and ‘unpaired’ cerebral ganglia at 1 h after training. See also Supplementary Table S1.

Our previous work established that the implicit memory trace resulting from classical conditioning in *Lymnaea* is both acquired and stored in the neural circuit located in two distinct areas of the brain, namely the buccal and cerebral ganglia (hereafter ‘learning ganglia’)^26^. Also, we have reported on the construction of sncRNA cDNA libraries from the ‘learning ganglia’ dissected from trained snails to address the question of whether miRNAs play a role in the consolidation of LTM after single-trial classical conditioning. Next Generation Sequencing of these libraries has revealed several LTM-related miRNAs including miR-1175^15^ (Fig. 1a). Our bioinformatic analysis predicted that this miRNA could bind to a complementary target sequence located within a lncRNA known as the *Lym-NOS1AS* NAT (Fig. 1b). Furthermore, our calculations of free energy of the Lym-miR-1175/*Lym-NOS1AS* duplex formation (ΔGduplex, − 14.6 kcal/mol) and total free energy difference (ΔΔG = ΔGduplex – ΔGopen, − 9 kcal/mol) by the PITA algorithm^27^ indicated a high binding affinity between these two ncRNAs. Consequently, these observations bring up the intriguing possibility that Lym-miR-1175 regulates *Lym-NOS1AS* NAT and, through this, is involved in NO-dependent LTM.

If Lym-miR-1175 plays a role in LTM, it would be expected to see specific changes in its expression induced by conditioning. Therefore, using RT-qPCR we quantified the expression level of Lym-miR-1175 in the buccal (BG) and the cerebral (CG) ganglia dissected from conditioned snails at 1 h, 2 h, 4 h and 6 h after the training trial (Fig. 1c). We revealed precisely timed and targeted learning-induced changes in the expression level of Lym-miR-1175. Specifically, we found that Lym-miR-1175 was up-regulated (by approximately 4 times) in the CG at 1 h after conditioning (Fig. 1d). We detected no significant alterations in the expression of Lym-miR-1175 in the BG (Fig. 1e).

### Lym-miR-1175 downregulates the non-coding NOS-related long natural antisense transcript *Lym-NOS1AS*

**Fig. 2:**
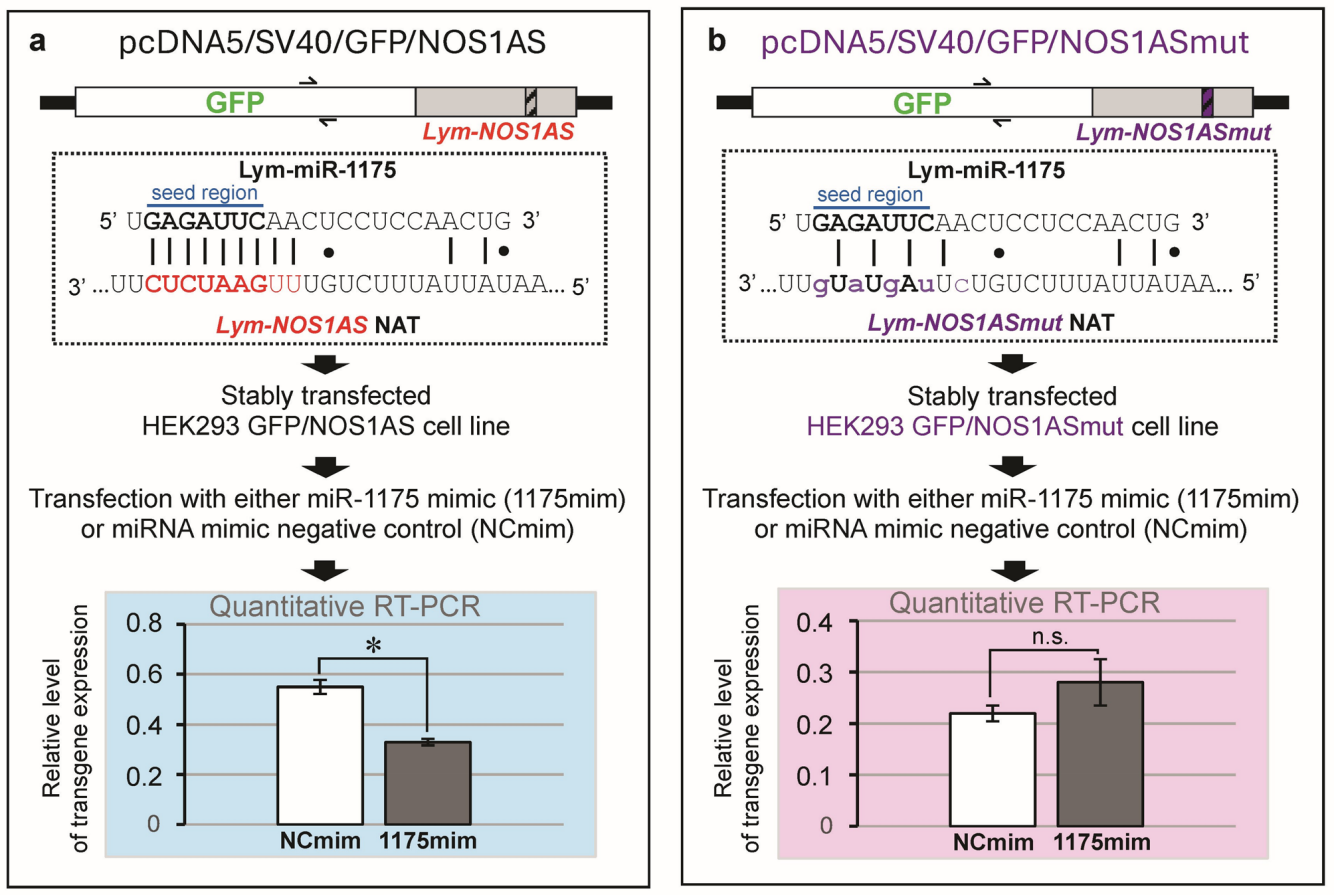
Lym-miR-1175 downregulates *Lym-NOS1AS* NAT. (**a**) Schematic diagram of the experiments showing that overexpression of miR-1175 in the stably transfected HEK293 GFP/NOS1AS cell line results in the decreased level of the *GFP/Lym-NOS1AS* transgene expression. Hatched box shows the putative binding site within the *Lym-NOS1AS*. It is important to note that the seed region of the Lym-miR-1175 exhibits 100% complementarity to the target site located within the *Lym-NOS1AS* (the non-Watson–Crick G–U base pairs are shown by dots). Asterisk indicates significant difference between the control (white bar, NCmim) and the experimental (grey bar, 1175mim) samples (Mann-Whitney U Test, z-score 2.50672, *p* = 0.012). (**b**) Schematic diagram of the experiments showing that overexpression of miR-1175 in stably transfected HEK293 GFP/NOS1ASmut cell line does not change the level of the *GFP/Lym-NOS1ASmut* transgene expression. Nucleotide substitutions in the *Lym-NOS1ASmut*, which lower the level of complementarity to the Lym-miR-1175, are shown by lowercase letters. The Mann-Whitney U Test reveals no significant difference between the control (white bar, NCmim) and the experimental (grey bar, 1175mim) samples. Of note, the whole procedure was run 5 times to enable statistics and all data in this figure are shown as means±SEM. See also Supplementary Table S2. The approximate positions of primers used in RT-PCR experiments are shown by half arrows.

Our suggestion that Lym-miR-1175 can specifically target the NOS-related NAT was based on the observation that the seed region of the Lym-miR-1175 exhibited 100% complementarity to the putative recognition site within the *Lym-NOS1AS* (Fig. 2a). To provide more direct evidence for the role of Lym-miR-1175 in the regulation of *Lym-NOS1AS*, we used the gain-of-function approach. Specifically, we have inserted a large fragment of the *Lym-NOS1AS* into the pcDNA5/SV40/GFP plasmid, downstream of a GFP ORF and upstream of a polyadenylation site (Fig. 2a). The recombinant plasmid was transfected into HEK293 cells and a cell line with stable expression of the *GFP*/*Lym-NOS1AS* was generated. The generated cells were then treated with either Lym-miR-1175 mimic or negative control mimic and the expression level of the *GFP/Lym-NOS1AS* transgene was measured by real-time RT-PCR. As shown on Fig. 2a, overexpression of miR-1175 results in the decreased level of the *GFP*/*Lym-NOS1AS* compared with the effect caused by the negative control mimic.

To further strengthen our idea that Lym-miR-1175 specifically binds to the target site within the *Lym-NOS1AS*, we employed a site-directed mutagenesis approach. Specifically, we made 5 nucleotide substitutions in the putative binding site. This lowered significantly the level of complementarity to the Lym-miR-1175 (Fig. 2b). We then generated a cell line with stable expression of the mutated variant of the *Lym-NOS1AS* (GFP/Lym-NOS1ASmut). Importantly, the overexpression of the miR-1175 in these cells did not significantly change the transgene expression (Fig. 2b). This supports strongly our hypothesis that Lym-miR-1175 specifically downregulates *Lym-NOS1AS* NAT.

### Lym-miRNA-1175 is required for single-trial induced LTM formation

**Fig. 3:**
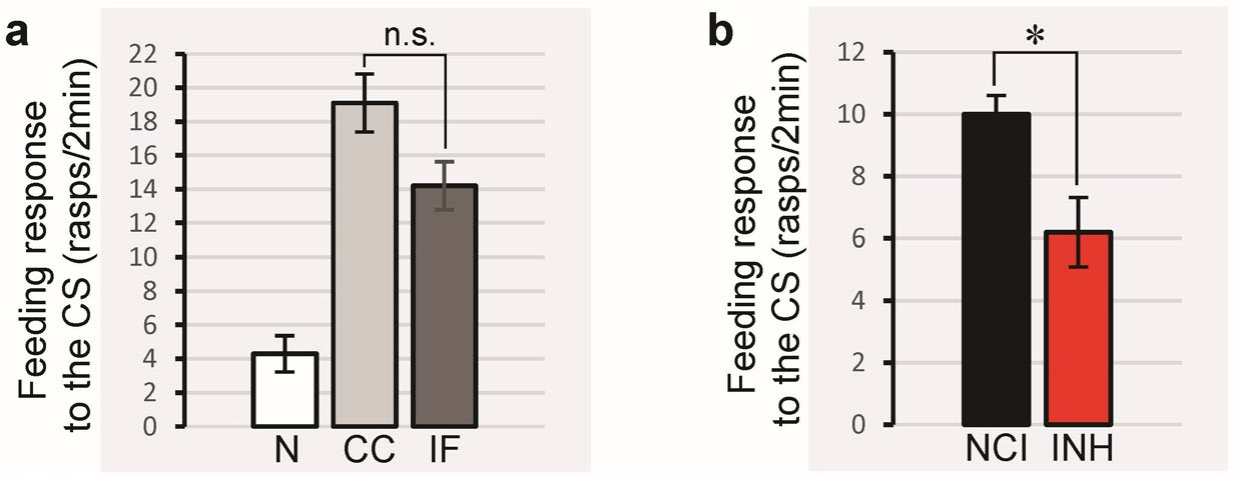
The role of Lym-miR-1175 in LTM. (**a**) Injection of snails with Invivofectamine does not impair LTM formation. Snails injected with Invivofectamine were trained and then tested for feeding response to the amyl acetate (CS) at 24 h after training. One-way ANOVA with post-hoc Tukey HSD test revealed no significant changes between the mean feeding responses of the Control Conditioned snails (light grey bar, CC, *n* = 16) and the conditioned animals injected with InvivoFectamine (dark grey bar, IF, *n* = 22, *p* = 0.11). Note that the feeding response of Naive animals (white bar, N, *n* = 17) is significantly lower than the response of the CC group (*n* = 16, *p* < 0.00001) and the IF group (*n* = 22, *p* < 0.001). (**b**) Blocking of Lym-miR-1175 impairs LTM after single-trial conditioning in vivo. All snails were tested for feeding response to the CS at 24 h after training. To test if the Lym-miR-1175 inhibitor down regulates LTM formation, we used the unpaired two-sample one-tailed t-test. It showed that the mean feeding response of the animals injected with the Lym-miR-1175 Inhibitor (red bar, INH) is significantly lower than the response of the snails injected with the Negative Control Inhibitor (black bar, NCI, *n* = 20, *p* = 0.035, t = 1.86). All data in these figures are shown as means ± SEM. See also Supplementary Table S3.

To investigate if Lym-miR-1175 is required for memory formation we employed an in vivo loss-of-function approach. First, in our control experiments, we examined whether the transfection reagent Invivofectamine causes any unwanted toxic/stressful reaction in our system. We made a direct comparison between the feeding responses to the CS of naïve animals, control conditioned animals and conditioned animals injected with Invivofectamine. Importantly, our results demonstrate that the control and Invivofectamine treated conditioned groups exhibit very similar conditioned responses (Fig. 3a). This proves that Invivofectamine does not impair LTM formation and, therefore, can be used in our further experiments. Consequently, in the second set of tests we compared the conditioned feeding responses of animals injected with either the miR-1175 inhibitor or the negative control inhibitor (Fig. 3b). We found that the injection of the miRNA-1175 inhibitor into the snails significantly suppressed LTM.

### Lym-miR-1175, *Lym‑NOS1AS* NAT and *Lym‑NOS1* mRNA are co‑expressed in a key neuron of the memory network

**Fig. 4:**
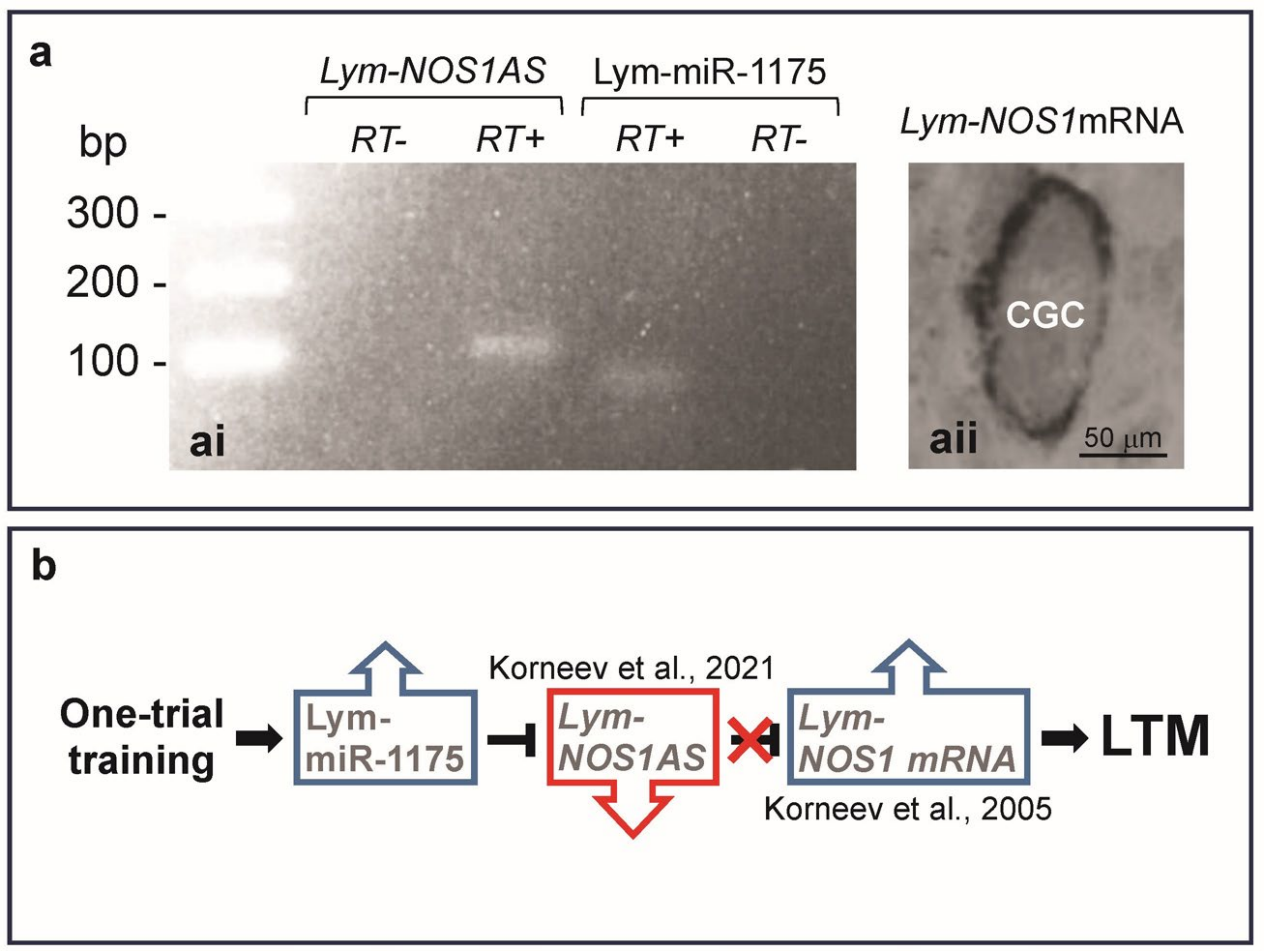
Co-expression of Lym-miR-1175, *Lym-NOS1AS* NAT and *Lym-NOS1* mRNA in the cerebral giant cell is in favour of the existence of a new pathway involved in LTM formation. (**a**) Lym-miR-1175, *Lym-NOS1AS* NAT and *Lym-NOS1* mRNA are co-expressed in the cerebral giant cell (CGC). (**ai**) The results of RT-PCRs conducted on RNA extracted from isolated CGCs indicate the presence of *Lym-NOS1AS* NAT and Lym-miR-1175. Of note, PCR products of the expected sizes were detected using standard DNA electrophoresis in a mini gel, and their identity was confirmed by cloning and sequencing. The ‘RT−’ lanes represent the outcome of the control experiments in which reverse transcriptase was omitted. See also Supplementary Fig. 1. (**aii**) In situ hybridization reveals the presence of *Lym-NOS1* mRNA in the cytoplasm of the CGC. (**b**) Schematic diagram shows the proposed role of the Lym-miR-1175/*Lym-NOS1AS*/*Lym-NOS1* pathway in single-trial induced LTM.

Our findings that the Lym-miR-1175 supresses the *Lym-NOS1AS* NAT and is required for LTM are of great interest because they indicate that the interaction between the two different types of ncRNAs is involved in memory formation. Furthermore, previously we and others demonstrated that NOS-related NATs (e.g., *Lym-NOS1AS*) act as negative regulators of NO production in the brain most likely through down-regulation of the NOS-encoding mRNAs^16–18^. Of note, NO has been shown to be necessary for memory formation after single-trial conditioning in *Lymnaea*^23^. But the proposed interplay between Lym-miR-1175, *Lym-NOS1AS* and *Lym‑NOS1* mRNA is possible only if these three RNAs co-exist in a neuron (Fig. 4a). To address this important point, we exploited the advantage of the existence of the pair of CGCs in the *Lymnaea* brain, which are identified neurons with a well-established role in the long-term conditioned feeding response. Using a combination of in situ hybridization and RT-PCR, we demonstrate that Lym-miR-1175, *Lym-NOS1AS* and *Lym-NOS1* mRNA are indeed co-expressed in the CGCs (Fig. 4a). Thus, collectively, these findings support our idea that Lym-miR-1175/*Lym-NOS1AS*/*Lym-NOS1* interaction is required for single-trial induced LTM formation.

## Discussion

Our everyday life instructs that not all our experiences culminate in the formation of durable long-lasting memories. This is due to the existence of the molecular mechanisms that provide inhibitory constraints on LTM formation. Therefore, single-trial induced LTMs require this suppressive effect to be immediately removed following the learning event. Recent findings indicate that miRNAs play important roles in this process by targeting specific messenger RNAs that encode memory-suppressor proteins^7,8,15^. However, little is known about whether such ‘memory enhancer’ miRNAs can interact with lncRNAs, especially those that are classified as long NATs. In the current paper, we aim to fill this gap by presenting experimental data uncovering the role of the interaction between a miRNA and a NOS-related long NAT in NO-dependent LTM (Fig. 4b).

First, we report on the discovery of the LTM-related Lym-miR-1175, the seed region of which is 100% complementary to a potential recognition site within the *Lym-NOS1AS* long NAT. Furthermore, we provide evidence that one-trial learning causes very specific temporal and spatial change in Lym-miR-1175 expression. More specifically, we found that Lym-miR-1175 was up-regulated in the cerebral ganglia at 1 h after training. Notably, this targeted up-regulation of Lym-miR-1175 agrees with the previous findings, which showed that the cerebral ganglia perform most of the NO-dependent information processing during memory consolidation^19^. Thus, the results of our quantitative analysis are in favour of our proposition that Lym-miRNA-1175 is involved in single-trial induced LTM formation, possibly through the interaction with *Lym-NOS1AS*.

Second, we understand that what is currently known about the interaction between miRNAs and long NATs is rather limited and, therefore, our prediction that Lym-miR-1175 targets *Lym-NOS1AS* long NAT, although intriguing and inspiring, requires robust experimental evidence. Therefore, we used a combination of gain-of-function and site-directed mutagenesis experiments and demonstrated that Lym-miR-1175 indeed down-regulates the *Lym-NOS1AS* NAT. But NOS-related NATs were shown to regulate NOS gene expression^17–22^ and NOS, in its turn, produces NO, which plays an important role in the early stages of memory formation. For example, in *Lymnaea*, there is an obligatory requirement for NO signalling in single-trial induced LTM for a continuous period of about 6 h post-training^23^. Thus, collectively, our data raise the exciting possibility that Lym-miR-1175, through negative regulation of *Lym-NOS1AS* NAT, acts as an important memory promoter molecule.

Third, in order to further strengthen our reasoning for the role of the Lym-miR-1175 in LTM formation, we carried out behavioural experiments with intact snails. Specifically, we injected animals with either the miR-1175 inhibitor or an unrelated negative control inhibitor. We found that the injection of miR-1175 inhibitor had a significant suppressive effect on memory formation, supporting the importance of Lym-miR-1175 for LTM. However, we have to mention that blockage of Lym-miR-1175 did not completely suppress LTM, likely because of the existence of other powerful memory constraints^15^.

Finally, we would like to draw attention to some interesting correlations between training-induced expression profiles of Lym-miR-1175, *Lym-NOS1AS* and *Lym-NOS1* mRNA in the cerebral ganglia. Indeed, whereas the current study reveals up-regulation of Lym-miR-1175, our previous work showed that *Lym-NOS1AS* is down-regulated in the same ganglia after conditioning^16^. Furthermore, we can deduce from our earlier studies that this down-regulation of *Lym-NOS1AS* is followed by the up-regulation of *Lym-NOS1* mRNA^19^. These findings suggest the existence of a new pathway for LTM formation, which depends on the interaction between Lym-miR-1175, *Lym-NOS1AS* NAT and *Lym-NOS1* mRNA. In this pathway, Lym-miR-1175 appears to act as a critical trigger, removing the *Lym-NOS1AS* memory constraint. In turn, this promotes *Lym-NOS1* mRNA expression, ensuring the production of NO, which is essential for LTM formation (Fig. 4b). We understand that this interplay between different classes of RNAs involved in the regulation of NO signalling can occur only if Lym-miR-1175, *Lym-NOS1AS* and *Lym-NOS1* mRNA co-exist in a neuron or neurones with a key role in memory formation. Importantly, our in situ hybridisation and single-cell RT-PCR experiments confirmed that these three RNAs are indeed co-expressed in the CGCs, which fulfil the above criterion^24^.

In conclusion, while a number of miRNAs regulating other types of lncRNAs (for example, intergenic lncRNAs) have been identified^28–34^, the interplay between miRNAs and long NATs remains an under-investigated area^35]– [36^. Consequently, we anticipate that the results presented here will contribute to a better understanding of the molecular neurobiology of single-trial induced LTM and will also generate new insights into complex relationships between miRNAs and lncRNAs.

## Methods

### Experimental animals

 Specimens of *Lymnaea stagnalis* were raised in the breeding facility of the University of Sussex, where they were kept in 20–22 °C copper free water under 12 h light/dark cycle. The snails were fed on lettuce and a vegetable-based fish food.

### Single-trial conditioning protocol

 Reward conditioning was performed using a method based on a previously published protocol^23^. Briefly, snails were randomly assigned to experimental (paired) and control (unpaired) groups. The experimental group was exposed to a solution of amyl acetate (CS, 0.04%) and immediately after that to a sucrose solution (US, 0.67%). Animals from the control group were exposed to the CS and to the US, separated by an interval of 1 h. A randomly chosen subset of 20 animals from each group was retained and tested for LTM formation at 24 h after the paired and unpaired trials.

### Quantitative real-time RT-PCR

RNA preparations were obtained from the cerebral and the buccal ganglia dissected at 1 h, 2 h, 4 h and 6 h after training from conditioned (paired) and control (unpaired) snails (*n* = 20 in each group, collected in 5 tubes each containing 4 either BG or CG) by means of the Absolutely RNA miRNA kit (Agilent). The purified RNA samples were treated with DNase TURBO (Ambion) to remove any traces of DNA. Each purified RNA sample was divided into two equal parts. The first parts were used to quantify Lym-miR-1175 by employing the Custom TaqMan™ Small RNA Assay 4,398,987/CS1RUKG (Life Technologies). RNAs from the second part were copied into cDNAs using the iScript cDNA synthesis kit (Bio-Rad). The cDNAs produced were amplified and analysed on the Mx3000P real-time cycler (Stratagene) using the SYBR Green qPCR Master mix (Biotool). We used primers 5′-AAGGGACATTACACAGAGG-3′ and 5′-GTGTCAGTTGGAATCCTTG-3′ for the detection of β-tubulin (endogenous reference). The expression level of Lym-miR-1175, normalized to the endogenous reference and relative to a calibrator, was calculated as 2^−ΔΔCT 37^.

### Gain-of-function experiments

*Plasmid vector construction.* The CMV⁄TetO2 promoter was removed from the pcDNA5⁄FRT⁄TO plasmid (ThermoFisher Scientific) and replaced with the SV40 promoter. Also, the GFP-encoding gene was inserted downstream of the CMV promoter. The new plasmid vector was named pcDNA5/SV40/GFP.

*Construction of recombinant plasmids expressing the Lym-NOS1AS.* The forward (5’-ACTTGCTCGAGTGTGTGCAGTTACTTGAG-3’) and reverse (5’- ACTTGCTCGAGATTTACCACAATGGCTCAG-3’) PCR primers were used to amplify a 455 bp fragment of *Lym-NOS1AS* containing the putative binding site for the Lym-miR-1175. The product was digested with XhoI, purified and inserted into the pcDNA5/SV40/GFP. The resulting recombinant plasmid was named pcDNA5/SV40/GFP/NOS1AS. In parallel experiments, the *Lym-NOS1AS* containing mutated bunding site cTtAgTaTg (lowercase letters show the difference from the wild type binding site) exhibiting a low level of complementarity to Lym-miR-1175 was created using PCR-based site-directed mutagenesis. The mutated construction was ligated with the pcDNA5/SV40/GFP, and the resulted recombinant plasmid was named pcDNA5/SV40/GFP/NOS1ASmut.

*Stable cell line generation.* The Flp-In™ 293 T-REx cell lines (ThermoFisher Scientific) were cultured in DMEM supplemented with 10% foetal bovine serum, 2 mM L-glutamate and 1% penicillin-streptomycin.

pOG44 Flp-recombinase expression vector (Invitrogen) was co-transfected into the Flp-In 293 T-REx cells with either the pcDNA5/SV40/GFP/NOS1AS or the pcDNA5/SV40/GFP/NOS1ASmut. Hygromycin (150 mg/ml) was added and single clones of stable transfectants were individualized and selected after 14 days of incubation. These resulting stable cell lines were termed HEK293 GFP/NOS1AS and HEK293 GFP/NOS1ASmut.

*Transfection of miR-1175 mimic in stably transfected HEK293 GFP/NOS1AS and HEK293 GFP/NOS1ASmut cell lines.* The same protocol was used for the HEK293 GFP/NOS1AS and HEK293 GFP/NOS1ASmut cell lines. Briefly, one day before transfection, 10,000 stably transfected HEK293 cells were plated in a 4 cm^2^ dish containing MEM supplemented with 15% foetal bovine serum. On the day of transfection, we diluted the mirVana miR-1175 mimic MC15379 (#4464066, Ambion) in Opti-MEM Reduced Serum Medium for a final concentration of 120 nM. In a separate tube, Lipofectamine 2000 (Invitrogen) was diluted 50 times using the same medium. We then mixed 100 µl of the miR-1175 mimic and 100 µl of the diluted Lipofectamine 2000 and incubated the mixture for 20 min at room temperature. We added 200 µl of the miR-1175 mimic-Lipofectamine complex to the dish containing stably transfected HEK293 cells. The cells were incubated at 37 °C in a CO_2_ incubator for 24 h until they were ready for quantitative real-time RT-PCR analysis. Of note, in the control experiment, the miR-1175 mimic was replaced with the mirVana miRNA Mimic Negative Control 1 (#4464058, Ambion).

*Quantification of transgene expression.* RNA was extracted from the transfected cells by means of Absolutely RNA Microprep Kit (Agilent), then copied into cDNA using the iScript cDNA synthesis kit (Bio-Rad) and the cDNA produced was amplified using the SYBR Green qPCR Master mix (Biotool). Primers 5’-TGGCCGACAAGCAGAAGA-3’ and 5’-GTGTTCTGCTGGTAGTGG-3’ were used to quantify transgene expression. Primers 5’-AAGGTGAAGGTCGGAGTC-3’ and 5’-GTAAACCATGTAGTTGAGGTC-3’ were used to detect the *GAPDH* gene expression. The expression level of the transgene, normalized to the endogenous reference (*GAPDH*) and relative to a calibrator, was calculated as 2^−ΔΔCT^.

Notably, the whole procedure was run 5 times to enable statistics.

### Silencing of Lym-miR-1175 in vivo

 To investigate if Invivofectamine 2 (Life Technologies) has a nonspecific side effect on LTM formation, we injected the reagent into the haemolymph of 20 snails before single-trial training. The feeding response of the injected snails to the CS was tested 24 h after training and compared to the feeding responses of naïve snails and snails subjected to a standard procedure of classical single-trial conditioning.

To silence Lym-miR-1175 in vivo, we used the *mir*VANA miRNA inhibitor (#4464086, Ambion). Specifically, the inhibitor complex containing 50 µl (17 µg) of the miRNA-1175 inhibitor, 50 µl of complexation buffer and 100 µl of Invivofectamine 2 was prepared according to the manufacturer’s protocol (Life Technologies). The complex was incubated at 50 °C for 30 min and then purified using the Float-A-Lyzer G2 dialysis device according to the manufacturer’s protocol (Spectrum Medical Laboratories). The volume was adjusted to 2.5 ml with the saline buffer and 100 µl of the final solution was injected into the haemolymph of 20 snails 48 h before training. A control group of snails (*n* = 20) was subjected to the same procedure but animals from this group were injected with the Negative Control Inhibitor (#4464076, Ambion), which is a random sequence anti-miR molecule that has been validated to produce no identifiable effects on known miRNA function.

All experimental and control animals were tested with the CS 24 h after training to evaluate LTM formation. An individual feeding score was calculated by subtracting the number of rasps elicited by water from that elicited by the addition of the CS.

### RT-PCR on the cerebral giant cells

 The cell bodies of CGCs were identified and dissected from the CNS of *Lymnaea* as described previously^19^. Total RNA extracted from the CGCs by means of the Absolutely RNA miRNA kit (Agilent) was split into separate halves. The first half was used to detect Lym-miR-1175 with the aid of the Custom TaqMan™ Small RNA Assay 4,398,987/CS1RUKG (Life Technologies). The second half was used to identify *Lym-NOS1AS*. Specifically, the purified RNA was copied into cDNAs using the iScript cDNA synthesis kit (Bio-Rad) and the cDNA produced was amplified using the SYBR Green qPCR Master mix (Biotool) in the presence of 5′-GTAATAAGCGCATTTGCATAC-3′ and 5′-CCTGGTGTGAAGCTGATC-3′ primers. The identity of the PCR products was confirmed by cloning and sequencing.

###  In situ hybridization

 Detection of *Lym-NOS1* mRNA in frozen sections of *Lymnaea* CNS by in situ hybridization was performed as previously described^38^. The labelled probe (5′-CACAGGA(AC)GGTATGGTGTTCT-3′) was prepared using the DIG Oligonucleotide Tailing Kit (Roche) according to the manufacturer’s protocol.

### Statistical analysis

In both the behavioural and molecular experiments comparisons between two independent groups (e.g., unpaired and paired) were carried out using unpaired two-tailed Student’s t-tests or Mann-Whteney U-tests. Welch’s correction was used when the samples had unequal variances. A one-tailed t-test was performed when we were testing for a change in one direction only. Differences among more than two groups were tested using ANOVA followed by Tukey’s multiple comparisons. The differences were considered statistically significant at *p* < 0.05.

## Supplementary Information

Below is the link to the electronic supplementary material.

Supplementary Material 1

Supplementary Material 2

Supplementary Material 3

Supplementary Material 4

## Data Availability

All data generated during this study are included in the published article and its supplementary information files.

## References

[1] Kandel, E. R. The molecular biology of memory storage: A dialogue between genes and synapses. *Science* **294**, 1030–1038. 10.1126/science.1067020 (2001). 11691980 10.1126/science.1067020

[2] Brown, R. & Kulik, J. Flashbulb memories. *Cognition* **5**, 73–99. 10.1016/0010-0277(77)90018-X (1977).

[3] Sierra, M. & Berrios, G. E. Flashbulb memories and other repetitive images: A psychiatric perspective. *Compr. Psychiatry*. **40**, 115–125. 10.1016/s0010-440x(99)90115-3 (1999). 10080258 10.1016/s0010-440x(99)90115-3

[4] Lee, S. W., O’Doherty, J. P. & Shimojo, S. Neural computations mediating one-shot learning in the human brain. *PLoS Biol.* **13**, e1002137. 10.1371/journal.pbio.1002137 (2015). 25919291 10.1371/journal.pbio.1002137 PMC4412411

[5] Barry, G. Integrating the roles of long and small non-coding RNA in brain function and disease. *Mol. Psychiatry*. **19**, 410–416. 10.1038/mp.2013.196 (2014). 24468823 10.1038/mp.2013.196

[6] Cech, T. R. & Steitz, J. A. The noncoding RNA revolution-trashing old rules to Forge new ones. *Cell* **157**, 77–94. 10.1016/j.cell.2014.03.008 (2014). 24679528 10.1016/j.cell.2014.03.008

[7] McNeill, E. & Van Vactor, D. MicroRNAs shape the neuronal landscape. *Neuron* **75**, 363–379. 10.1016/j.neuron.2012.07.005 (2012). 22884321 10.1016/j.neuron.2012.07.005 PMC3441179

[8] Rajasethupathy, P. et al. A role for neuronal PiRNAs in the epigenetic control of memory-related synaptic plasticity. *Cell* **149**, 693–707. 10.1016/j.cell.2012.02.057 (2012). 22541438 10.1016/j.cell.2012.02.057 PMC3442366

[9] Busto, G. U., Guven-Ozkan, T., Fulga, T., Van Vactor, D. & Davis, R. MicroRNAs that promote or inhibit memory formation in *Drosophila melanogaster*. *Genetics* **200**, 569–580. 10.1534/genetics.114.169623 (2015). 26088433 10.1534/genetics.114.169623 PMC4492380

[10] Capitano, F. et al. MicroRNAs modulate Spatial memory in the hippocampus and in the ventral striatum in a region-specific manner. *Mol. Neurobiol.* **53**, 4618–4630. 10.1007/s12035-015-9398-5 (2016). 26307611 10.1007/s12035-015-9398-5

[11] Harris, C. A., Passaro, P. A., Kemenes, I., Kemenes, G. & O’Shea, M. Sensory driven multi-neuronal activity and associative learning monitored in an intact CNS on a multielectrode array. *J. Neurosci. Methods*. **186**, 171–178. 10.1016/j.jneumeth.2009.11.0144 (2010). 19941897 10.1016/j.jneumeth.2009.11.014

[12] Kemenes, G. Molecular and cellular mechanisms of classical conditioning in the feeding system of *Lymnaea*. In *Invertebrate Learning and Memory* (eds. Menzel, R. & Benjamin, P.) 251–264 (Elsevier, 2013).

[13] Korneev, S. A. et al. Axonal trafficking of an antisense RNA transcribed from a pseudogene is regulated by classical conditioning. *Sci. Rep.* **3**, 1027. 10.1038/srep01027 (2013). 23293742 10.1038/srep01027 PMC3537157

[14] Kemenes, G. Dynamic molecular mechanisms of memory consolidation after single-trial food-reward classical conditioning in *Lymnaea.* In *Memory Consolidation* (eds. Ito, E. & Sakakibara, M.) 127–140 (Nova, 2015).

[15] Korneev, S. A. et al. A CREB2-targeting MicroRNA is required for long-term memory after single-trial learning. *Sci. Rep.* **8**, 3950. 10.1038/s41598-018-22278-w (2018). 29500383 10.1038/s41598-018-22278-w PMC5834643

[16] Korneev, S. A., Garaliene, J., Taylor, G., Kemenes, I. & Kemenes, G. Time dependent differential regulation of a novel long non-coding natural antisense RNA during long-term memory formation. *Sci. Rep.* **11**, 3594. 10.1038/s41598-021-83190-4 (2021). 33574420 10.1038/s41598-021-83190-4 PMC7878882

[17] Korneev, S. A., Park, J-H. & O’Shea, M. Neuronal expression of neural nitric oxide synthase (nNOS) protein is suppressed by an antisense RNA transcribed from an NOS pseudogene. *J. Neurosci.* **19**, 7711–7720. 10.1523/JNEUROSCI.19-18-07711.1999 (1999). 10479675 10.1523/JNEUROSCI.19-18-07711.1999 PMC6782476

[18] Robb, G. B. et al. Post-transcriptional regulation of endothelial nitric-oxide synthase by an overlapping antisense mRNA transcript. *J. Biol. Chem.* **279**, 37982–37996. 10.1074/jbc.M400271200 (2004). 15234981 10.1074/jbc.M400271200

[19] Korneev, S. A. et al. Timed and targeted differential regulation of NOS and AntiNOS genes by reward conditioning leading to long-term memory formation. *J. Neurosci.* **25**, 1188–1192. 10.1523/JNEUROSCI.4671-04.2005 (2005). 15689555 10.1523/JNEUROSCI.4671-04.2005 PMC6725956

[20] Fish, J. E. et al. Hypoxia-inducible expression of a natural cis-antisense transcript inhibits endothelial nitric-oxide. *J. Biol. Chem.* **282**, 15652–15666. 10.1074/jbc.M608318200 (2007). 17403686 10.1074/jbc.M608318200

[21] Korneev, S. A. et al. Novel noncoding antisense RNA transcribed from human anti-NOS2A locus is differentially regulated during neuronal differentiation of embryonic stem cells. *RNA* **14**, 2030–2037. 10.1261/rna.1084308 (2008). 18820242 10.1261/rna.1084308 PMC2553742

[22] Korneev, S. A. et al. A novel long non-coding natural antisense RNA is a negative regulator of Nos1 gene expression. *Sci. Rep.* **5**, 11815. 10.1038/srep11815 (2015). 26154151 10.1038/srep11815 PMC4495418

[23] Kemenes, I., Kemenes, G., Andrew, R. J., Benjamin, P. R. & O’Shea, M. Critical time-window for NO–cGMP-dependent long-term memory formation after one-trial appetitive conditioning. *J. Neurosci.* **22**, 1414–1425. 10.1523/JNEUROSCI.22-04-01414.2002 (2002). 11850468 10.1523/JNEUROSCI.22-04-01414.2002 PMC6757551

[24] Kemenes, I. et al. Role of delayed nonsynaptic neuronal plasticity in long-term associative memory. *Curr. Biol.* **16**, 1269–1279. 10.1016/j.cub.2006.05.049 (2006). 16824916 10.1016/j.cub.2006.05.049

[25] Hermann, P. M. et al. Impairment of long-term associative memory in aging snails (*Lymnaea stagnalis*). *Behav. Neurosci.* **121**, 1400–1414. 10.1037/0735-7044.121.6.1400 (2007). 18085894 10.1037/0735-7044.121.6.1400

[26] Benjamin, P. R. Distributed network organization underlying feeding behavior in the mollusk *Lymnaea*. *Neural Syst. Circ.* **2**, 4. 10.1186/2042-1001-2-4 (2012). 10.1186/2042-1001-2-4 PMC3350398 22510302

[27] Kertesz, M., Iovino, N., Unnerstall, U., Gaul, U. & Segal, E. The role of site accessibility in MicroRNA target recognition. *Nat. Genet.* **39**, 1278–1284. 10.1038/ng2135 (2007). 17893677 10.1038/ng2135

[28] Braconi, C. et al. MicroRNA-29 can regulate expression of the long non-coding RNA gene MEG3 in hepatocellular cancer. *Oncogene* **30**, 4750–4756. 10.1038/onc.2011.193 (2011). 21625215 10.1038/onc.2011.193 PMC4292930

[29] Yoon, J-H. et al. LincRNA-p21 suppresses target mRNA translation. *Mol. Cell.* **47**, 648–655. 10.1016/j.molcel.2012.06.027 (2012). 22841487 10.1016/j.molcel.2012.06.027 PMC3509343

[30] Wang, Y. et al. Endogenous MIRNA sponge lincRNA-RoR regulates Oct4, Nanog, and Sox2 in human embryonic stem cell self-renewal. *Dev. Cell.* **25**, 69–80. 10.1016/j.devcel.2013.03.002 (2013). 23541921 10.1016/j.devcel.2013.03.002

[31] Chiyomaru, T. et al. Genistein inhibits prostate cancer cell growth by targeting Mir-34a and oncogenic HOTAIR. *PLoS ONE*. **8**, e70372. 10.1371/journal.pone.0070372 (2013). 23936419 10.1371/journal.pone.0070372 PMC3731248

[32] Leucci, E. et al. microRNA-9 targets the long non-coding RNA MALAT1 for degradation in the nucleus. *Sci. Rep.* **3**, 2535. 10.1038/srep02535 (2013). 23985560 10.1038/srep02535 PMC3756333

[33] Wang, H. et al. MALAT1/miR-101-3p/MCL1 axis mediates cisplatin resistance in lung cancer. *Oncotarget* **9**, 7501–7512. 10.18632/oncotarget.23483 (2017). 29484127 10.18632/oncotarget.23483 PMC5800919

[34] Wang, Z. et al. Long non-coding RNA taurine upregulated gene 1 (TUG1) downregulation constrains cell proliferation and invasion through regulating cell division cycle 42 (CDC42) expression via miR-498 in esophageal squamous cell carcinoma cells. *Med. Sci. Monit.* **26**, e919714. https//doi.org10.12659/MSM.919714 (2020). 10.12659/MSM.919714 PMC7077061 32139664

[35] Hansen, T. B. et al. miRNA-dependent gene Silencing involving Ago2‐mediated cleavage of a circular antisense RNA. *EMBO J.* **30**, 4414–4422. 10.1038/emboj.2011.359 (2011). 21964070 10.1038/emboj.2011.359 PMC3230379

[36] Mu, X. et al. Long noncoding RNA TMPO-AS1 promotes lung adenocarcinoma progression and is negatively regulated by miR-383-5p. *Biomed. Pharmacother*. **125**, 109989. 10.1016/j.biopha.2020.109989 (2020). 32062549 10.1016/j.biopha.2020.109989

[37] Pfaffl, M. W. A new mathematical model for relative quantification in real-time RT-PCR. *Nucleic Acids Res.* **29**, 2002–2007. 10.1093/nar/29.9.e45 (2001). 10.1093/nar/29.9.e45 PMC55695 11328886

[38] Korneev, S. A. et al. Molecular characterization of NOS in a mollusc: Expression in a giant modulatory neuron. *J. Neurobiol.* **35**, 65–76 (1998). https://doi.org/10.1002/(SICI)1097-4695(199804)35:1<65::AID-NEU6>3.0.CO;2-9 9552167 10.1002/(sici)1097-4695(199804)35:1<65::aid-neu6>3.0.co;2-9

