# Supplementary material for: miRNA-1175 downregulates a long non-coding natural antisense RNA and promotes long term memory
Source: Sci Rep. 2025 Nov 5;15:38718. doi: 10.1038/s41598-025-22550-w (PMC12589416; doi:10.1038/s41598-025-22550-w)
Supplement: Supplementary file 1 — Supplementary Material 1 [file 41598_2025_22550_MOESM1_ESM.pdf]

Relative expression level of Lym-miR-1175 in the Buccal Ganglia dissected at 1h, 2h, 4h and 6h after training

Paired, P

Unpaired, UP

Sample	Ct miRNA1175	Ct TUBULIN	DCt	DDCt	RelExpr	**average**	St Er
**1h P**							
N1	26,9	24	2,9	-1,2	2,3	**2,2**	0,19
N2	25,6	22,9	2,7	-1,4	2,6		
N3	27,6	24,5	3,1	-1	2		
N4	28,5	25,2	3,3	-0,8	1,7		
**1h UP**							
N1	24,8	21,9	2,9	-1,2	2,3	**3,1**	0,52
N2	24,5	22,2	2,3	-1,8	3,5		
N3	25,5	22,4	3,1	-1	2		
N4	25,2	22,6	2,6	-1,5	2,8		
N5	27,8	26	1,8	-2,3	4,9		
**2h P**							
N1	26.янв	24	2,5	-2,1	4,3	**2,5**	0,48
N2	26,2	22,9	3,3	-1,3	2,5		
N3	31	27,7	3,3	-1,3	2,5		
N4	28,4	24,6	3,8	-0,8	1,7		
N5	32,9	29	3,9	-0,7	1,6		
**2h UP**							
N1	24,1	21,4	2,7	-1,9	3,7	**3,7**	0,28
N2	26,8	23,8	3	-1,6	3		
N3	26,9	24,3	2,6	-2	4		
N4							
N5	25,3	22,8	2,5	-2,1	4,3		

Sample	Ct miRNA1175	Ct TUBULIN	DCt	DDCt	RelExpr	**average**	St Er
**4h P**							
N1	27	24,1	2,9	-1,2	2,3	**2**	0,4
N2	26,3	23,9	2,4	-1,7	3,2		
N3	25	21,1	3,9	-0,2	1,1		
N4	24,4	21,4	3	-1,1	2,1		
N5	25,7	21,8	3,9	-0,2	1,1		
**4h UP**							
N1							
N2	23,2	22,6	0,6	-3,5	11,3	**4,9**	2,18
N3	26,1	23,1	3	-1,1	2,1		
N4	26,1	23,1	3	-1,1	2,1		
N5	23,4	21,4	2	-2,1	4,3		
**6h P**							
N1	26,7	25	1,7	-2,4	5,3	**9,6**	2,26
N2	23,9	21,8	2,1	-2	4		
N3	23,8	23,7	0,1	-4	16		
N4	25,4	24,6	0,8	-3,3	9,8		
N5	24,1	23,7	0,4	-3,7	13		
**6h UP**							
N1	23,7	22	1,7	-2,4	5,3	**5,5**	0,94
N2	24,2	22,1	2,1	-2	4		
N3	24,9	23	1,9	-2,2	4,6		
N4	23,9	23	0,9	-3,2	9,2		
N5	25,4	23,5	1,9	-2,2	4,6		

Relative expression level of Lym-miR-1175 in the Cerebral Ganglia dissected at 1h, 2h, 4h and 6h after training

Paired, P

Unpaired, UP

Sample	Ct miR1175	Ct TUBULIN	DCt	DDCt	Rel Expr	**average**	St Er
**1h P**							
N1	22,4	21,7	0,7	-1,6	3	**4,1**	0,94
N2	21,7	21,5	0,2	-2,1	4,3		
N3	21,6	21,2	0,4	-1,9	3,7		
NA	22,2	20,8	1,4	-0,9	1,9		
NC	21,4	22	-0,6	-2,9	7,5		
**1h UP**							
N1	22,6	21,2	1,4	-0,9	1,9	**1,2**	0,31
N2	23,4	20,5	2,9	0,6	0,6		
N3	23	21,7	1,3	-1	2		
N4	23,5	20,3	3,2	0,9	0,5		
N5	22,3	20,3	2	-0,3	1,2		
**2h P**							
N1	23,1	21,8	1,3	-1	2	**2,5**	0,59
N2	24,6	23,7	0,9	-1,4	2,6		
N3	23,8	21,3	2,5	0,2	0,9		
N4	22,5	22,4	0,1	-2,2	4,5		
N5	22,8	22	0,8	-1,5	2,8		
**2h UP**							
N1	22,9	21,6	1,3	-1	2	**2,9**	1,16
N2	21,7	20,2	1,5	-0,8	1,7		
N3	21,8	20,4	1,4	-0,9	1,9		
N4	22,2	22,8	-0,6	-2,9	7,5		
N5	22,2	20,3	1,9	-0,4	1,3		

Sample	Ct miR1175	Ct TUBULIN	DCt	DDCt	Rel Expr	**average**	St Er
**4h P**							
N1	21,9	22,3	-0,4	-2,7	6,5	**5,9**	0,39
N2	22,2	22,3	-0,1	-2,4	5,3		
N3	23,1	23	0,1	-2,2	4,6		
N4	22,2	22,6	-0,4	-2,7	6,5		
N5	22,3	22,6	-0,4	-2,7	6,5		
**4h UP**							
N1	25,6	25	0,6	-1,7	3,2	**9**	1,66
N2	21,6	22,4	-0,8	-3,1	8,6		
N3	22,4	23,8	-1,4	-3,7	13		
N4	22,9	23,8	-0,9	-3,2	9,2		
N5	21,7	22,9	-1,2	-3,5	11,3		
**6h P**							
N1	21,9	22,2	-0,3	-2,5	5,6	**4,9**	0,92
N2	22	22,4	-0,4	-2,7	6,5		
N3	23	24,4	-1,4	-3,7	1,3		
N4	21,7	21,9	-0,2	-2,5	5,6		
N5	22,2	22,4	-0,2	-2,5	5,6		
**6h UP**							
N1	22,2	23,8	-1,6	-3,9	14,9	**6,1**	2,35
N2	22,1	22,4	-0,3	-2,6	6,1		
N3	21,7	21,7	0	-2,3	4,9		
N4	21,7	21,1	0,6	-1,7	3,2		
N5	21,8	19,9	1,9	-0,4	1,3		
