# Supplementary material for: miRNA-1175 downregulates a long non-coding natural antisense RNA and promotes long term memory
Source: Sci Rep. 2025 Nov 5;15:38718. doi: 10.1038/s41598-025-22550-w (PMC12589416; doi:10.1038/s41598-025-22550-w)
Supplement: Supplementary file 2 — Supplementary Material 2 [file 41598_2025_22550_MOESM2_ESM.pdf]

The results of qRT-PCR experiments showing that overexpression of miR-1175 in the stably transfected HEK293 GFP/NOS1AS cell line results in the decreased level of the *GFP/Lym-NOS1AS* transgene expression

Transfection with the negative control mimic (NCmim)

Experiment 1

Target	Ct (dRn)	AV	DCt	DDCt	**Rel Expr**
GAPDH	17,8	17,9			
	17,91				
	17,89				
transgene	19,91	19,8	1,9	1,1	**0,47**
	19,85				
	19,8				

Experiment 2

Target	Ct (dRn)	AV	DCt	DDCt	**Rel Expr**
GAPDH	19,78	19,8			
	19,75				
	19,89				
transgene	21,38	21,4	1,6	0,8	**0,57**
	21,49				
	21,33				

Experiment 3

Target	Ct (dRn)	AV	DCt	DDCt	**Rel Expr**
GAPDH	18,2	18,2			
	18,28				
	18,19				
transgene	20,01	20	1,8	1	**0,5**
	19,93				
	19,97				

Experiment 4

Target	Ct (dRn)	AV	DCt	DDCt	**Rel Expr**
GAPDH	20,18	20,2			
	20,19				
	20,18				
transgene	21,7	21,7	1,5	0,7	**0,61**
	21,76				
	21,77				

Experiment 5

Target	Ct (dRn)	AV	DCt	DDCt	**Rel Expr**
GAPDH	19,23	19,3			
	19,28				
	19,31				
transgene	20,77	20,8	1,5	0,7	**0,61**
	20,8				
	20,76				

Summary

	Rel Expr	**average**	St Error
Exp1	0,47	**0,55**	0,03
Exp2	0,57		
Exp3	0,5		
Exp4	0,61		
Exp5	0,61		

Transfection with the miR-1175 mimic (1175mim)

Experiment 1

Target	Ct (dRn)	AV	DCt	DDCt	**Rel Expr**
GAPDH	18,75	18,8			
	18,87				
	18,8				
transgene	20,85	20,9	2,1	1,8	**0,29**
	21,02				
	20,75				

Experiment 2

Target	Ct (dRn)	AV	DCt	DDCt	**Rel Expr**
GAPDH	19,2	19,2			
	19,22				
	19,29				
transgene	21	21	1,8	1,5	**0,35**
	20,96				
	21,03				

Experiment 3

Target	Ct (dRn)	AV	DCt	DDCt	**Rel Expr**
GAPDH	19,49	19,5			
	19,53				
	19,53				
transgene	21,38	21,3	1,8	1,5	**0,35**
	21,24				
	21,26				

Experiment 4

Target	Ct (dRn)	AV	DCt	DDCt	**Rel Expr**
GAPDH	18,96	19			
	19				
	18,97				
transgene	20,73	21,6	2,6	1,6	**0,33**
	21,2				
	22,81				

Experiment 5

Target	Ct (dRn)	AV	DCt	DDCt	**Rel Expr**
GAPDH	19,29	19,4			
	19,4				
	19,41				
transgene	21,15	21,2	1,8	1,5	**0,35**
	21,15				
	21,18				

Summary

	Rel Expr	average	St Error
Exp1	0,29	0,33	0,01
Exp2	0,35		
Exp3	0,35		
Exp4	0,33		
Exp5	0,35		

The results of qRT-PCR experiments showing that overexpression of miR-1175 in the stably transfected HEK293 GFP/NOS1ASmut cell line does not change the *GFP/Lym-NOS1AS* transgene expression

Transfection with the negative control mimic (NCmim)

Experiment 1

Target	Ct (dRn)	AV	DCt	DDCt	**Rel Expr**
GAPDH	18,6	18,59			
	18,59				
	18,57				
transgene	19,03	19,05	0,46	2,36	**0,19**
	19,08				
	19,04				

Experiment 2

Target	Ct (dRn)	AV	DCt	DDCt	**Rel Expr**
GAPDH	18,78	18,69			
	18,74				
	18,54				
transgene	19,02	19	0,31	2,21	**0,22**
	18,95				
	19,03				

Experiment 3

Target	Ct (dRn)	AV	DCt	DDCt	**Rel Expr**
GAPDH	18,86	18,8			
	18,76				
	18,78				
transgene	18,83	18,9	0,1	2	**0,25**
	18,99				
	18,87				

Experiment 4

Target	Ct (dRn)	AV	DCt	DDCt	**Rel Expr**
GAPDH	18,62	18,68			
	18,75				
transgene	18,96	19,02	0,34	2,24	**0,21**
	19,2				
	18,89				

Experiment 5

Target	Ct (dRn)	AV	DCt	DDCt	**Rel Expr**
GAPDH	18,53	18,35			
	18,31				
	18,2				
transgene	18,84	18,74	0,39	2,29	**0,2**
	18,67				
	18,72				

Summary

	Rel Expr	**average**	St Error
Exp1	0,19	**0,21**	0,01
Exp2	0,22		
Exp3	0,25		
Exp4	0,21		
Exp5	0,2		

Transfection with the miR-1175 mimic (1175mim)

Experiment 1

Target	Ct (dRn)	AV	DCt	DDCt	**Rel Expr**
GAPDH	18,45	18,47			
	18,52				
	18,44				
transgene	18,61	18,59	0,12	2,02	**0,25**
	18,58				
	18,58				

Experiment 2

Target	Ct (dRn)	AV	DCt	DDCt	**Rel Expr**
GAPDH	18,7	18,54			
	18,45				
	18,47				
transgene	18,72	18,79	0,25	2,15	**0,22**
	18,84				
	18,81				

Experiment 3

Target	Ct (dRn)	AV	DCt	DDCt	**Rel Expr**
GAPDH	20,08	19,94			
	19,86				
	19,88				
transgene	18,9	19	-0,94	0,96	**0,51**
	18,96				
	19,16				

Experiment 4

Target	Ct (dRn)	AV	DCt	DDCt	**Rel Expr**
GAPDH	19,07	19,17			
	19,15				
	19,28				
transgene	19,18	19,21	0,04	1,94	**0,26**
	19,16				
	19,28				

Experiment 5

Target	Ct (dRn)	AV	DCt	DDCt	**Rel Expr**
GAPDH	18,48	18,48			
	18,5				
	18,47				
transgene	18,57	18,56	0,08	1,98	**0,25**
	18,59				
	18,52				

Summary

	Rel Expr	**average**	St Error
Exp1	0,25	**0,3**	0,05
Exp2	0,22		
Exp3	0,51		
Exp4	0,26		
Exp5	0,25		
