# Supplementary material for: miRNA-1175 downregulates a long non-coding natural antisense RNA and promotes long term memory
Source: Sci Rep. 2025 Nov 5;15:38718. doi: 10.1038/s41598-025-22550-w (PMC12589416; doi:10.1038/s41598-025-22550-w)
Supplement: Supplementary file 3 — Supplementary Material 3 [file 41598_2025_22550_MOESM3_ESM.pdf]

In this experiment we investigated if Invivofectamine has a nonspecific side effect on LTM formation

Three groups of animals were used:

Naïve (N). This group was not exposed to either the CS or US

Control Conditioned (CC). This group was subjected to a standard procedure of classical single-trial conditioning

Invivofectamine (IF). This group was injected with IF ibefore single-trial training

An individual feeding score was calculated by subtracting the number of rasps (counted for 2 min) elicited by water from that elicited by the addition of the CS.

Naïve (N)

WATER	CS	SCORE	average	St Error
3	2	-1	4,3	1,5
2	14	12		
11	16	5		
2	0	-2		
0	10	10		
0	1	1		
0	15	15		
6	18	12		
13	19	6		
4	7	3		
3	9	6		
7	13	6		
5	4	-1		
1	2	1		
11	1	-10		
2	11	9		
0	1	1		

Control Conditioned (CC)

WATER	CS	SCORE	average	St Error
1	25	24	19,1	1,8
0	18	18		
1	19	18		
0	25	25		
4	33	29		
0	19	19		
0	28	28		
0	27	27		
0	22	22		
0	12	12		
10	31	21		
0	17	17		
0	17	17		
0	5	5		
0	6	6		
0	18	18		

Invivofectamine (IF)

WATER	CS	SCORE	average	St Error
0	7	7	14,2	1,7
2	24	22		
4	18	14		
0	22	22		
1	18	17		
6	21	15		
3	39	36		
1	25	24		
0	5	5		
2	3	1		
4	15	11		
0	8	8		
0	7	7		
0	19	19		
6	25	19		
4	22	18		
2	10	8		
0	5	5		
0	13	13		
3	15	12		
4	19	15		
2	17	15		

In this experiment we compared the conditioned feeding responses of animals injected with either the miR-1175 inhibitor or the negative control inhibitor

Two groups of animals were used:

Inhibitor Negative Control (NC). Animals were trained 48h after injection with the miRNA inhibitor negative control

miR-1175 Inhibitor (INH). Animals were trained 48h after injection with the Lym-miR-1175 inhibitor

An individual feeding score was calculated by subtracting the number of rasps (counted for 2 min) elicited by water from that elicited by the addition of the CS.

Inhibitor Negative Control (NC)

WATER	CS	SCORE	average	St Error
2	5	3	**10**	1,4
3	6	3		
0	22	22		
1	14	13		
4	10	6		
11	17	6		
5	18	13		
8	25	17		
0	19	19		
4	24	20		
4	16	12		
0	17	17		
0	12	12		
4	5	1		
0	4	4		
0	8	8		
3	12	9		
0	5	5		
6	22	16		
0	2	2		
2	8	6		
0	6	6		

miR-1175 inhibitor (INH)

WATER	CS	SCORE	average	St Error
7	19	12	**6,2**	1,5
11	1	-10		
0	7	7		
0	1	1		
0	8	8		
0	0	0		
6	22	16		
4	8	4		
5	12	7		
0	10	10		
5	7	2		
2	11	9		
6	14	8		
19	32	13		
4	6	2		
0	0	0		
0	0	0		
0	13	13		
3	19	16		
