# Supplementary material for: miRNA-1175 downregulates a long non-coding natural antisense RNA and promotes long term memory
Source: Sci Rep. 2025 Nov 5;15:38718. doi: 10.1038/s41598-025-22550-w (PMC12589416; doi:10.1038/s41598-025-22550-w)
Supplement: Supplementary file 4 — Supplementary Material 4 [file 41598_2025_22550_MOESM4_ESM.pdf]

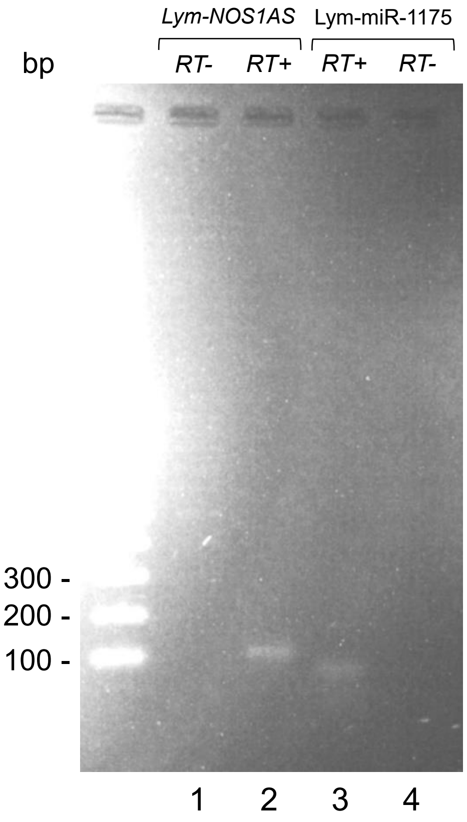

The results of RT-PCRs conducted on RNA extracted from isolated CGCs indicate the presence of *Lym-NOS1AS* NAT (lane 2) and Lym-miR-1175 (lane 3). PCR products of the expected sizes were detected using standard DNA electrophoresis, and their identity was confirmed by cloning and sequencing. The ‘RT−’ lanes (lanes 1 and 4) represent the outcome of the control experiments in which reverse transcriptase was omitted.
